# Assessment of the role of ageing and non-ageing factors in death from non-communicable diseases based on a cumulative frequency model

**DOI:** 10.1038/s41598-017-08539-0

**Published:** 2017-08-15

**Authors:** Liu Hui

**Affiliations:** Department of Clinical Immunology, Dalian Medical University, Dalian, 116044 China

## Abstract

To quantify the effects of ageing and non-ageing factors, a characterization of the effects of ageing, genetic, and exogenous variables on 12 major non-communicable diseases was evaluated using a model assessing cumulative frequency of death and survival by age group from dead and surviving populations based on mortality statistics. Indices (0–1) of the roles of ageing (ARD), genetics (GRD) and exogenous (ERD) variables in deaths due to disease were established, and the sum of ARD, GRD and ERD was 1 (value of each indices was <1). Results showed that ageing plays an important role in death from chronic disease; exogenous factors may contribute more to the pattern of chronic disease than genetic factors (ARD, GRC and ERD were 0.818, 0.058 and 0.124 respectively for all non-communicable diseases). In descending order, ERD for non-communicable diseases were breast cancer, leukaemia, cancer of the cervix uteri and uterus, liver cancer, nephritis and nephropathy, stomach cancer, lung cancer, diabetes, cerebrovascular disease, coronary heart disease, COPD, and Alzheimer’s disease, while a smaller ERD indicated a tendency of natural death. An understanding of the aforementioned complex relationships of specific non-communicable diseases will be beneficial in designing primary prevention measures for non-communicable diseases in China.

## Introduction

Modern non-communicable diseases or chronic diseases, such as cardiovascular disease, cancer, and obstructive pulmonary disease, have become the leading causes of death worldwide^1–3^. It is generally accepted that both genetic and environmental factors are related to mechanisms of chronic diseases; here, “genetic factors” are intrinsically hereditary, excluding epigenetic variables; “environmental factors” are all environmental factors including aspects of the social and the natural environment such as stress, lack of physical activity and environmental pollutants. Certain extrinsic risk factors, such as smoking, being overweight, and hyperlipidaemia, may lead to chronic diseases, though they are not necessarily associated with any particular disease^4–6^. Hence, the assessment and control of such risk factors is complicated. Moreover, the incidence of non-communicable diseases tends to increase with age; therefore, a discussion of the aforementioned risk factors shall highlight the association of non-communicable diseases with ageing. Here, “ageing” results in changes in individual capacity to withstand exogenous physical, social and psychological (psychosocial) stressors with increased age. Although the ageing effect could be controlled through manipulation of the exogenous variables that are the causative agents and genetic factors, the role of ageing, genetic and exogenous variables could still be assessed in a certain disease because ageing, genetic and exogenous variables could be stable for a particular population. A better understanding of these associations will be beneficial in developing a theoretical basis for strategies for the prevention and control of non-communicable diseases.

The incidence and progression of non-communicable diseases are affected by various factors, which can be stratified into ageing and non-aging variables. Non-ageing variables include exogenous variables and genetic factors. Age is one of the most important risk factors associated with death due to non-communicable diseases^7–9^. Quantitative analysis of the role of ageing in disease can be used to speculate regarding the effect of non-ageing factors. We propose a role for ageing in death due to disease (ARD) based on the difference in cumulative frequency by age in both dead and surviving groups (cumulative frequency model) to evaluate the effect of ageing and non-ageing factors.

The first step towards understanding a selection of non-communicable diseases is the determination of the non-communicable disease characteristics that may be associated with risk factors. Therefore, it is necessary to quantitatively evaluate the effects of risk factors such as age, genetics and extrinsic variables on non-communicable diseases. In the present study, we used death statistics for all age groups from the census data for Mainland China and established methods for assessing the risk factors for death from non-communicable diseases. Simultaneously, we also selected significant non-communicable diseases on the basis of the quantitative effects of ageing, genetics and extrinsic factors and compared the features of these diseases.

## Methods

### Original data

The raw data were obtained from the dataset of China’s National Disease Mortality Surveillance System for 2011, which was edited by the Chinese Centre for Disease Control and Prevention and published by the People’s Medical Publishing House^10^. The data for this particular year were obtained from over 77 million people living in urban and rural areas in the eastern, central and western regions of China and representing approximately 6% of China’s total population. The proportion of people surveyed from urban and rural areas in eastern, central and western regions was similar to that of the whole Chinese population. Further details are provided in Tables 1 and 2. The population count at midnight on July 1, 2011, represented the surviving population.

**Table 1:** Population distribution obtained by the surveillance system in China.

Gender	Region	Eastern	Central	Western
Male	Urban	7,337,456	5,217,568	3,061,909
	Rural	7,941,533	8,544,613	7,277,543
Female	Urban	7,124,417	5,116,332	2,990,504
	Rural	7,781,578	8,176,687	6,826,338

**Table 2:** Age-stratified number of deaths from major chronic diseases in 2011 in China^10^.

Age	Non-communicable diseases													All-cause	Survival
	A	B	C	D	E	F	G	H	I	J	K	L	All		
0-	0	2	0	0	0	26	0	0	0	1	0	7	1522	5440	933752
1-	0	9	0	0	0	76	2	0	0	3	0	5	640	2217	3392878
5-	0	4	0	0	0	65	2	0	0	7	0	12	339	1123	4012131
10-	0	4	2	0	0	77	1	0	0	9	0	15	343	1032	3868981
15-	5	12	11	0	2	122	10	1	38	46	2	38	696	2093	5785176
20-	22	40	16	4	4	162	14	1	122	97	10	60	1125	3562	6809078
25-	37	87	38	12	30	130	27	1	163	146	14	80	1346	3535	5784092
30-	85	225	89	38	46	123	47	3	252	258	38	92	2008	4429	5344198
35-	137	576	255	102	122	114	72	4	561	629	59	164	4138	7425	7071179
40-	377	1140	636	220	189	188	121	5	1154	1417	164	235	8254	12534	7225187
45-	628	1632	1194	323	293	185	262	11	1905	2683	322	298	13431	17851	6043321
50-	914	1747	1707	302	278	172	300	21	2125	3364	596	289	15838	19040	5461624
55-	1538	2616	3116	435	372	272	575	41	3326	5692	1353	399	26155	30206	5080008
60-	2002	2459	3637	272	274	225	843	54	4475	7799	2368	435	32051	35591	3392477
65-	2049	2113	3808	214	225	228	1010	112	5283	10111	3671	491	36959	40115	2600448
70-	2419	2094	4564	171	216	211	1298	219	8348	14850	6835	577	51582	55292	2103617
75-	2427	1820	4583	160	195	240	1511	472	11083	19169	10036	688	63759	68269	1474549
80-	1606	1261	2978	125	126	149	1123	771	12571	18865	11556	540	61853	67429	775419
>85	911	686	1594	67	71	67	801	1174	15570	16666	11492	418	58886	68294	238363

Diseases classified by ICD-10 code: A: stomach cancer (C16); B: liver cancer (C22); C: lung cancer (C33-34); D: breast cancer (C50); E: cervix uteri and uterine cancer (C53-55); F: leukaemia (C91-95); G: diabetes (E10-14); H: Alzheimer’s disease (F01-03, G30, 31); I: coronary heart disease (I20-25); J: cerebrovascular disease (I60-69); K: chronic obstructive pulmonary disease (J40-44); L: nephritis and nephropathy (N00-19); All: total non-communicable diseases (C00-97, D00-48, 55–89, E03-07, 10–16, 20–34, 65–88, F01-99, G06-98, H00-61, 68–93, I00-99, J30-98, K00-92, N00-64, 75–98, L00-98, M00-99, Q00-99); All-cause: death from all causes; Survival: number of population at midnight on July 1, 2011 according to survey.

The underlying causes of death were classified according to the International Classification of Diseases (ICD-10 codes) in order to determine mortality statistics^11^. The raw data are presented in Table 2.

### The role of ageing in deaths caused by chronic disease

**Figure 1:**
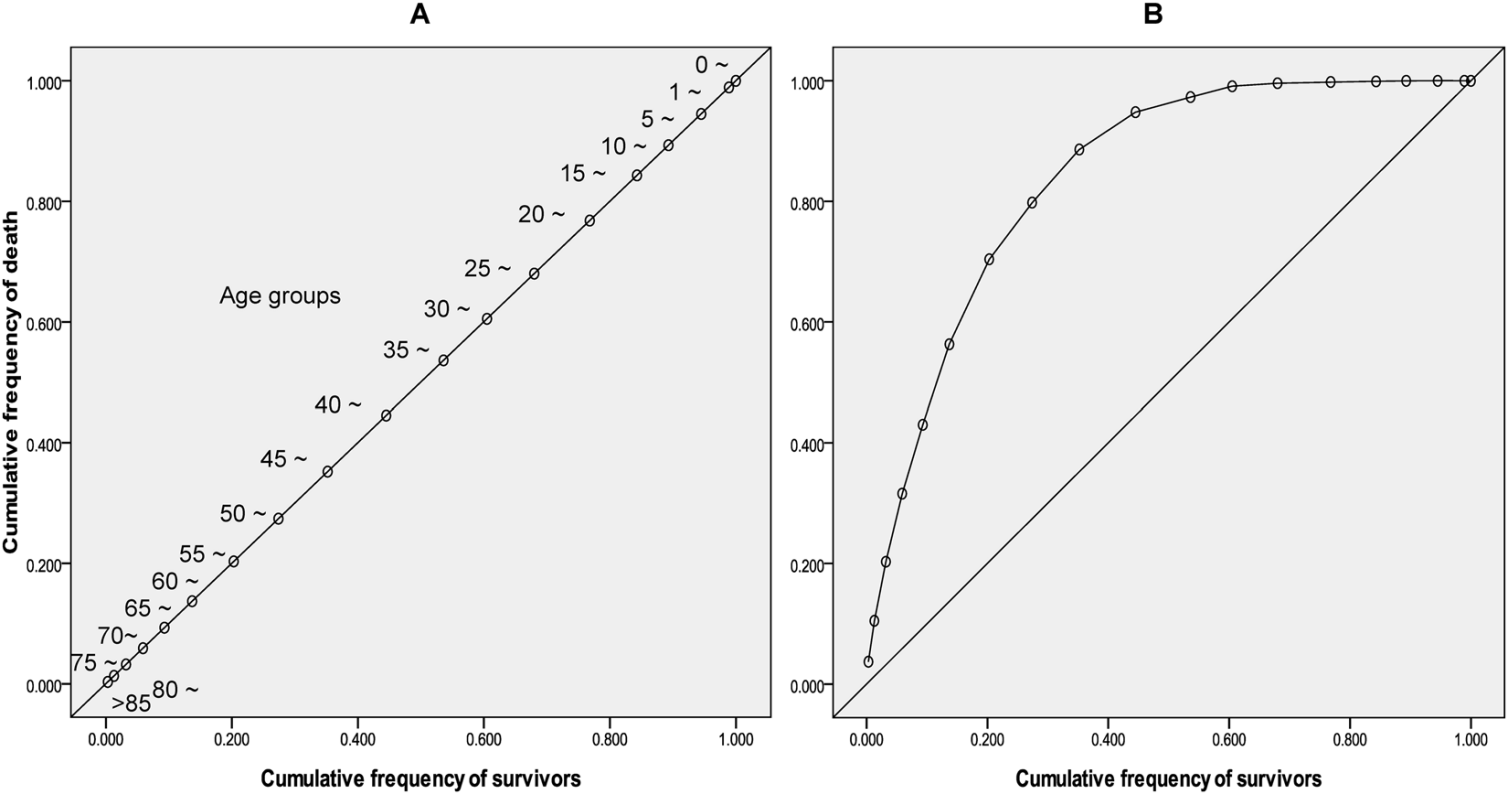
Scatter plot showing the cumulative frequency of survival and the cumulative frequency of death by age group. (**A**) age was not associated with death; (**B**) age had an impact on death from a disease.

The observed population was ranked by age in descending order. If age was not associated with death, the cumulative frequency of survival by age group ought to be consistent with the cumulative frequency of death by age group. The cumulative frequency of survival was plotted on the x-axis, and the cumulative frequency of death in the population by age group was plotted on the y-axis. A scatter plot of the 45-degree diagonal segment was obtained (Fig. 1A). If age had an impact on death, the cumulative frequency of survival was different from that of the population who had died, and the scatter plot curve would be distant from the 45-degree diagonal line (Fig. 1B). The area enclosed by the curve and the 45-degree diagonal line was interpreted as the role of ageing in death (ARD). Age has the greatest impact on death, with a value of 0.5. Therefore, the curve and the 45-degree diagonal area enclosed by the ratio of 0.5 defines the role of age in the quantitative value of death. This is how ARD is represented.

Receiver operating characteristics (ROC) analysis can be used to calculate ARD because there is a relationship between the area under the curve (AUC) from ROC analysis and the area enclosed by the curve and the 45-degree diagonal from the ARD analysis^12^. The x-axis and y-axis denote the cumulative frequency of survival and the population of those who died by age group, respectively. The AUC can represent the impact of age on death, and ARD can be calculated using the following equation:

1 $${\rm{ARD}}=({\rm{AUC}}-0.5)/0.5\quad {\rm{AUC}} > 0.5$$

The range of ARD was between 0 and 1. A high ARD indicated that disease mortality was mainly due to ageing, and the impact of non-ageing factors was less important.

ROC analysis was performed using SPSS for Windows, version 17.0. The difference in the AUC from 0.5 was considered statistically significant when the probability of a type I error was 0.05 or less.

### Role of non-age-related diseases resulting in death

Analysis of the role of non-age-related death (NARD) could include two factors: (1) genetic role in death (GRD) and (2) exogenous role in death (ERD). The basic concept of this analysis is to consider the age of onset as a genetic effect based on existing knowledge^13–15^. For complex diseases such as cancer, age of disease onset is generally thought to be related to a combination of influences related to the duration of exogenous exposures and genetic susceptibility; in effect, subjects with an inherited susceptibility may develop cancer at an earlier age when exposed to the same exogenous exposures^16–18^.

In the present study, the age cut-off at death for different diseases was quantified using ROC analysis, where the ages of people who died from a non-communicable disease were compared to the ages of those who survived over the same period. Mortality should increase markedly when a population exceeds the age cut-off at death for a particular disease; the component below age cut-off at death could be considered GRD, and the component above the age cut-off at death could be considered ERD for this disease.

NARD can be calculated from (1 − ARD). GRD and ERD can be calculated from the following equation:

2 $${\rm{GRD}}={\rm{NARD}}\times {\rm{Fd}}=(1-{\rm{ARD}})\times {\rm{Fd}}$$

3 $${\rm{ERD}}={\rm{NARD}}\times (1-{\rm{Fd}})=(1-{\rm{ARD}})\times (1-{\rm{Fd}})$$

where Fd represents the cumulative frequency of death in the low age cut-off group.

**Figure 2:**
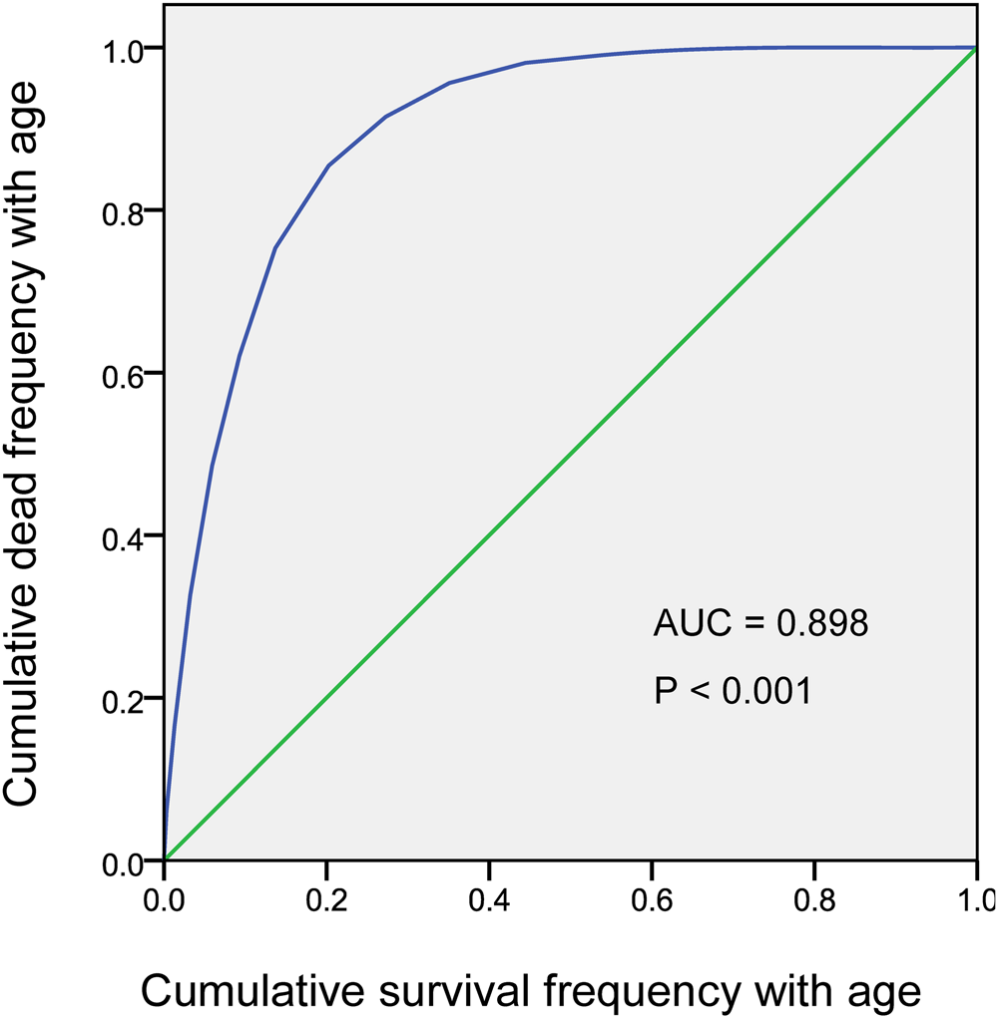
ROC analysis to calculate the age of deaths from stomach cancer as an example.

We used ROC analysis to calculate the ARD for stomach cancer as an example, which is shown in Fig. 2. The cumulative frequency of death (Fd) in the low age cut-off group was 0.145.

$$\,\begin{array}{rcl}{\rm{AUC}} & = & 0.898,\\ {\rm{ARD}} & = & (0.898-0.5)/0.5=0.796,\\ {\rm{NARD}} & = & (1-0.796)=0.204,\,\\ {\rm{GRD}} & = & 0.204\times 0.145=0.030\\ {\rm{ERD}} & = & 0.204\times (1-0.145)=0.174\\ {\rm{Total}}\,{\rm{role}} & = & 0.796+0.030+0.174=1.000\end{array}$$

## Results

The raw data and index of ARD, GRD and ERD for the stipulated non-communicable diseases are summarized in Table 3.

**Table 3:** Raw data and indices for ageing, genetic, and exogenous role in death caused by chronic diseases.

Diseases	AUC	Cut-off Age	Cumulative frequency		Index		
			Before cut-A	After cut-A	ARD	GRD	ERD
Stomach cancer	0.898	55.0	0.145	0.855	0.796	0.062	0.142
Liver cancer	0.842	45.0	0.113	0.887	0.684	0.136	0.180
Lung cancer	0.900	55.0	0.140	0.860	0.800	0.060	0.140
Breast cancer	0.805	45.0	0.154	0.846	0.610	0.147	0.243
Cervix uteri and uterine cancer	0.808	45.0	0.161	0.839	0.616	0.149	0.235
Leukaemia	0.671	55.0	0.508	0.492	0.342	0.299	0.359
Diabetes	0.915	55.0	0.107	0.893	0.830	0.052	0.118
Alzheimer’s disease	0.977	55.0	0.016	0.984	0.954	0.041	0.005
Coronary heart disease	0.934	60.0	0.144	0.856	0.868	0.040	0.092
Cerebrovascular disease	0.933	60.0	0.141	0.859	0.866	0.039	0.095
COPD	0.963	60.0	0.053	0.947	0.926	0.022	0.052
Nephritis and nephropathy	0.845	55.0	0.267	0.733	0.690	0.112	0.198
Non-communicable diseases	0.909	55.0	0.130	0.870	0.818	0.058	0.124
All-cause death	0.878	55.0	0.180	0.820	0.756	0.073	0.171

ARD, GRD and ERD represent the indices for ageing, genetic, and exogenous roles in death, respectively; AUC represents the area under the curve from ROC analysis; AUC: area under the curve; cut-A: cut-off age; COPD: chronic obstructive pulmonary disease.

A larger ARD indicated a tendency of natural death from ageing. In descending order, ARD for non-communicable diseases were Alzheimer’s disease, chronic obstructive pulmonary disease, coronary heart disease, cerebrovascular disease, diabetes, lung cancer, stomach cancer, nephritis and nephropathy, liver cancer, cancer of the cervix uteri and uterus, breast cancer, and leukaemia. There were seven non-communicable diseases with an ARD that exceeded all-cause death.

**Figure 3:**
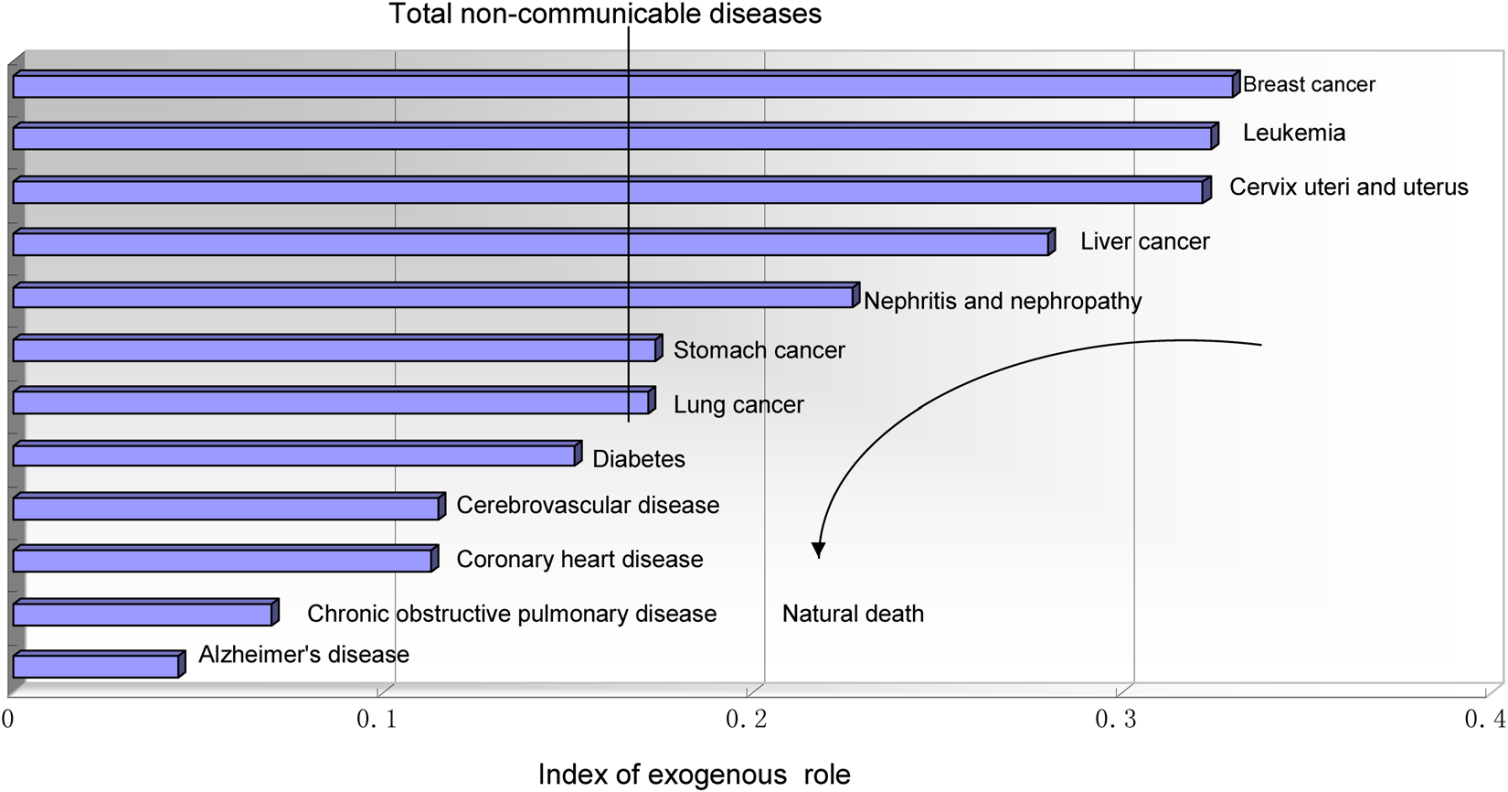
A tendency of natural death with weaker exogenous role in the diseases resulting in death.

A larger ERD indicated that a disease could be relatively well controlled. In descending order, ERD for non-communicable diseases were breast cancer, leukaemia, cancer of the cervix uteri and uterus, liver cancer, nephritis and nephropathy, stomach cancer, lung cancer, diabetes, cerebrovascular disease, coronary heart disease, chronic obstructive pulmonary disease, and Alzheimer’s disease. There were seven non-communicable diseases with an ERD that exceeded total non-communicable diseases. A smaller ERD also indicated a tendency of natural death, as shown in Fig. 3.

## Discussion

Non-communicable diseases are diverse. We mainly selected the non-communicable diseases with the greatest impact on expected survival while simultaneously considering the various types of non-communicable diseases. Tumours are complicated, and different tumours vary significantly in their prognosis and outcomes; therefore, tumours were mainly recorded for the various systems. In this study, 12 non-communicable diseases were included, accounting for 79.46% of the total number of non-communicable diseases. These included the main fatal diseases of the nervous, respiratory, cardiovascular, digestive, endocrine, urogenital, and blood systems.

The cumulative frequency model in this study was based on a comparison between deaths and surviving groups; thus, the influence of the constituent ratio of age in the surviving population should be eliminated. Ageing is a key predictor of mortality for non-communicable disease, and the results show that ageing plays a dominant role in the pattern of chronic disease, suggesting that a model including cumulative frequency of death and survival by age group from dead and surviving populations was reasonable.

It is important to evaluate the effects of ageing on non-communicable disease. We defined non-communicable disease when ARD exceeded all-cause death of ARD as chronic diseases of ageing. Seven diseases (Alzheimer’s disease, chronic obstructive pulmonary disease, coronary heart disease, cerebrovascular disease, diabetes, lung cancer, and stomach cancer) accounted for 89.72% of the non-communicable diseases included in this study. For these particular diseases, ageing appears to be a major precipitant of death, and the role of non-aging variables appears to be limited. Since ageing cannot be influenced, preventative medical care could be a key measure for prolonging survival time with these diseases.

In this study, “exogenous” roles are the causative agents affecting the incidence of non-communicable disease. Non-communicable disease causative agents can be classified as primary (non-medical) factors (such as psychosocial stressors) or secondary causative agents (such as risky behaviours including smoking, diet and obesity, infections, radiation, lack of physical activity, and environmental pollutants)^19–21^. When ERD for a disease exceeded ERD for total non-communicable disease, this disease was defined as exogenous-related chronic disease. Seven diseases (breast cancer, leukaemia, cancer of the cervix uteri and uterus, liver cancer, nephritis and nephropathy, stomach cancer, and lung cancer) accounted for 24.6% of the 12 non-communicable diseases. Since the non-aging factors leading to disease can be influenced to some extent, we suggest that this group of diseases could be prevented by reducing risk factors, including tobacco use, being overweight, a nutritionally inadequate diet, physical inactivity, alcohol consumption, sexually transmitted infections, air pollution and a number of other risk factors that are modifiable. Moreover, a significant aspect of this study is that we included mortality data from women affected by three gynaecological cancers, and these can disproportionately be affected by the exogenous factors associated with such cancers.

The roles of aging, genetics, and exogenous factors are closely linked to disease status^20, 22, 23^. Data in this study suggest that ageing plays an important role in chronic disease, indicating that a natural death was the main cause of death from chronic disease. Without considering role of ageing, exogenous factors could play more of a role in the pattern of chronic disease resulting in death than genetic factors. To an extent, this indicates that chronic disease could be both preventable and controllable. With improvements in general health and medical care, the survival time for those with chronic diseases will be extended, which may be the main reason for prolonged life expectancy in more developed societies^24^.

Notably, the ageing effect, which is counted in the model of chronic disease resulting in death, could also be controlled through manipulation of the exogenous variables that are the causative agents of disease; therefore, the role of exogenous variables in chronic disease using our model may be underestimated. However, we speculate that interaction among aging, genetics, and exogenous factors may be limitation^12^ and our comprehensive model for chronic disease is still valid because ageing is relatively uncontrollable. Therefore, to improve the general health of the population, we should attempt to delay the biological ageing effect, improve medical care and extend the survival time for those affected by disease, which is the cornerstone of prevention and control of non-communicable diseases. It is clear that an understanding of the aforementioned features of each disease group will be beneficial for the primary prevention of non-communicable diseases.

In summary, 12 diseases were investigated using a cumulative frequency model, and these 12 diseases accounted for 90% of the total number of deaths from non-communicable disease, indicating that individuals with non-communicable diseases tend to die from natural ageing. As exogenous factors are relatively modifiable, it may be more effective to establish prevention and control measures against exogenous-related chronic diseases and may also be more meaningful in improving the level of care while extending the survival of those affected by diseases of ageing beyond their normal life expectancy.

Limitations of this study are that the model for assessing the role of ageing and non-aging factors in death has not been widely used, therefore, the study can only be considered preliminary. China covers a broad geographic area and is undergoing significant regional changes with rapid socioeconomic development. Therefore, earlier data can only be considered as a reference. We propose that the model may provide a better understanding of the characteristics of each non-communicable disease using new systems or concepts. Further studies are warranted to develop a more available model with new data for the identification of novel targets that can be used to create more effective and personalized complex disease prevention strategies.
